# Whole Genome Sequencing Identifies a Missense Mutation in *HES7* Associated with Short Tails in Asian Domestic Cats

**DOI:** 10.1038/srep31583

**Published:** 2016-08-25

**Authors:** Xiao Xu, Xin Sun, Xue-Song Hu, Yan Zhuang, Yue-Chen Liu, Hao Meng, Lin Miao, He Yu, Shu-Jin Luo

**Affiliations:** 1 Peking-Tsinghua Center for Life Sciences, Laboratory of Genomic Diversity and Evolution, School of Life Sciences, Peking University, Beijing 100871, China

## Abstract

Domestic cats exhibit abundant variations in tail morphology and serve as an excellent model to study the development and evolution of vertebrate tails. Cats with shortened and kinked tails were first recorded in the Malayan archipelago by Charles Darwin in 1868 and remain quite common today in Southeast and East Asia. To elucidate the genetic basis of short tails in Asian cats, we built a pedigree of 13 cats segregating at the trait with a founder from southern China and performed linkage mapping based on whole genome sequencing data from the pedigree. The short-tailed trait was mapped to a 5.6 Mb region of Chr E1, within which the substitution c. 5T > C in the somite segmentation-related gene *HES7* was identified as the causal mutation resulting in a missense change (p.V2A). Validation in 245 unrelated cats confirmed the correlation between *HES7-*c. 5T > C and Chinese short-tailed feral cats as well as the Japanese Bobtail breed, indicating a common genetic basis of the two. In addition, some of our sampled kinked-tailed cats could not be explained by either *HES7* or the Manx-related *T-box*, suggesting at least three independent events in the evolution of domestic cats giving rise to short-tailed traits.

The majority of vertebrate species, with the remarkable exceptions of humans and apes, possess a visible tail throughout their lifespans. The animal tail is an important appendage to the torso and plays adaptive roles in locomotion, balance, communication, thermoregulation and even energy storage^1^. In vertebrates, tails vary dramatically in color, size, shape and mobility and represent different evolutionary histories, including multiple independent events of shortening or loss of the tail in distinct lineages. Understanding the genetic causes of intraspecific tail length polymorphism would be one essential step toward elucidating the mechanisms underlying the development and evolution of tails. In laboratory mice, genetic studies of axial skeleton development have identified multiple genes and mutations involved in caudal vertebra development that have pleiotropic effects on fertility, somitogenesis, and meiotic recombination, thus shedding light on vertebrate evolution^2^,^3^,^4^,^5^. However, given the tremendous variety of tail morphology found in animals, mice alone may not be able to capture the full spectrum of the evolutionary diversity of mammalian tails. Thus, exploration of non-model species is necessary to obtain a full understanding of the underlying mechanisms.

Abundant phenotypic variations have arisen in the domestic cat (*Felis catus*) since its Near Eastern origin of domestication approximately 10,000 years ago^6^. Notably divergent from its wild ancestor *F. silvestris*, domestic cats have proven to be an important animal model for studying genotype-phenotype relationships as well as a comparative biomedical model for various human disease homologs^7^,^8^,^9^,^10^,^11^,^12^. The domestic cat exhibits several tail trait variations, among which length polymorphism is the most prominent. Cats with shortened tails were first documented in Southeast Asia extending into southern China and probably occurred for a long time before the establishment of most modern cat breeds, as recorded in 1868 by Charles Darwin^13^: “Throughout an immense area, namely the Malayan archipelago, Siam, Pegu, and Burmah, all the cats have truncated tails about half the proper length, often with a sort of knot at the end.” In southern China, short-tailed cats are named “Qilin tail” (after a mythical creature known in Chinese and other East Asian cultures) and are traditionally considered to be a symbol of fortune and wealth^14^,^15^. In addition to the length reduction, the tails of these cats often show kinks, a trait also referred to as “kinked tail”^16^.

Short-tailed domestic cats are widespread in Southeast Asia and southern China and they exhibit variable shortening and kinks that can be generally classified into three categories: (1) “minor kink”, which describes a tail showing only a slight shortening (approximately 25 cm long) with kink at distal region (Fig. 1A); (2) “medium kink”, which describes a tail showing a prominent truncation (approximately 10–20 cm long) with kinks at the proximal and/or distal caudal region (Fig. 1B); and (3) “extreme kink”, which describes a bobtail with severe truncation (less than 10 cm long) and multiple kinks (Fig. 1C). Cats with “minor kink” or “medium kink” tails are more common than those with “extreme kink” tails. Despite the deformity, such shortened and kinked tails create little physiological deficit affecting survival and reproduction, as evident in the persistence and prevalence of the trait in both feral and house cats in Southeast and East Asia. Empirical breeding practices have shown that a kinked-tailed kitten is always born from at least one kinked-tailed parent and that some of its littermates, if not all, usually display variable tail length and kinks, thus suggesting that a short tail in Asian cats is a dominant trait caused by a mutation(s) with variable degrees of expressivity. However, despite the wide geographic distribution, long history of prevalence, and cultural importance of the short-tailed cat in Asia, its underlying genetic causes remain elusive.

Abnormal lengths of tails are also recognized in at least five modern cat breeds in the world, including the Japanese Bobtail, the Manx, the American Bobtail, the Pixie-Bob and the Kurilian Bobtail cats^7^,^17^. A previous genetic study has linked the short-tailed/tailless trait in the Manx and several bobtail cat breeds to mutations in the *T-box* gene, whose homozygotes or compound heterozygotes result in embryonic lethality^7^. The *T-box* gene encodes the T-box family transcriptional factor Brachyury, which is specifically expressed in notochord and early mesoderm cells, and it plays an essential role in mesoderm formation and notochord differentiation^18^. Mutations in *T-box* also cause tail deformities in laboratory mice and dogs^18^,^19^. However, it has been shown that the *T-box* gene is not associated with the short-tailed trait in the Japanese Bobtail, thus suggesting that at least one additional locus determines the tail length polymorphisms in domestic cats^7^.

Unlike other Europe- or America-origin short-tailed cats, the Japanese Bobtail is strictly selected for a delicate short tail and exhibits a fixed bobtail trait, thus suggesting genetic homogeneity at the causal locus^15^. The tail of the Japanese Bobtail is approximately 10 cm long when extended and does not exhibit significant length variation, and it is composed of multiple curves or kinks curling it into a pom-pom shape. A genetic cross between a Japanese Bobtail and a cat with normal tail length has demonstrated dominant inheritance of the bobtail locus, and the short-tailed progeny have been found to exhibit kinks and tail length variation^20^. Given the morphological and hereditary resemblance of the Japanese Bobtail to the short-tailed domestic cats in Southeast Asia and southern China, it is reasonable to propose that the two may share the same genetic basis, and the ancestral founder of the Japanese Bobtail breed may originally be from Southeast Asia or China.

In this study, we constructed a two-generation pedigree from a kinked-tailed feral female cat from southern China crossed with a normal-tailed male domestic cat. We performed genome-wide linkage mapping and whole genome sequencing on the basis of the pedigree and identified a missense substitution in *HES7* that was associated with the kinked/short-tailed trait in Southeast Asian and southern Chinese cats. These results provide new insights into domestic cat evolution and the origin of some modern cat breeds.

## Results

### *T-box* mutation survey in short-tailed cats from southern China

Because *T-box* was the most likely candidate gene, its coding exons were amplified and sequenced in 20 kinked-tailed and 20 wild-type cats from southern China. Four SNPs were detected, including three synonymous substitutions (c.270C > T, c.324G > A, c.345C > G) and one non-synonymous change (c.1079C > T) resulting in a threonine-to-methionine substitution at amino acid residue 360 (p.T360M). Although p.T360M occurs at a conserved site, this variant represents a common variant within the cat population in southern China and is not associated with the target locus (*p* = 1.00). None of the four frame-shift mutations known to cause taillessness or short tails in the Manx cat (c.998delT, c.998_1014dup17delGCC, c.1169delC and c.1199delC) were detected in these Asian short-tailed cats^7^, thus confirming that the genetic bases underlying the European- and Asian-origin short-tailed cats are different.

### Pedigree establishment and tail phenotyping

**Figure 2:**
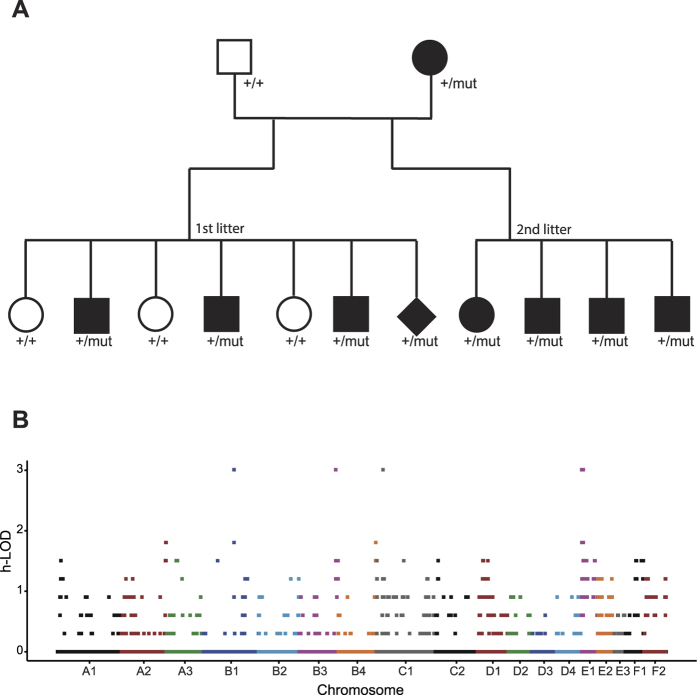
Linkage mapping of the kinked/short-tailed trait in Asian domestic cats. (**A**) a two-generation cat pedigree segregating for the kinked/short-tailed trait. Squares represent males, circles represent females, and a diamond represents the stillborn kitten. Solid icons represent kinked/short-tailed cats and open icons represent normal-tailed cats. Corresponding genotypes are given under the icon of each cat. (**B**) Manhattan plot summarizing the results of linkage analysis of the kinked/short-tailed trait on the basis of whole genome sequencing data from the 13 cats of the pedigree. SNPs within a 100 Kb window are clustered within one column.

A two-generation cat pedigree (N = 13) with segregation of the kinked/short-tailed trait was established in which the dam was suggested to be heterozygous for the kinked-tailed trait (medium kink) according to breeding records, and the sire was wild type. Two litters of 11 individuals from the F1 generation were born, including three normal- and eight short-tailed cats (Fig. 2A). Three of the eight mutant offspring belonged to the “minor kink” category, and the other five were categorized as “medium kink”. The tail trait segregation observed in this pedigree (8:3) indicated no significant deviation from the expected ratio under autosomal dominant inheritance (χ^2^ = 2.273, 1df; *p* = 0.1317). The observed morphological variations among the kinked-tailed offspring also suggested incomplete dominance of the mutation. This result is consistent with a previous genetic characterization study of the Japanese Bobtail^20^.

All individuals in the pedigree, except for one stillborn kitten, were subjected to radiography for precise examination of tail phenotypes. The wild-type cats had 22 caudal vertebrae with no sign of deformity (Table S1). The kinked-tailed dam and offspring all exhibited tail shortening, with the number of caudal vertebrae ranging from 14 to 21, and they had one to four caudal hemivertebrae (Table S1). In addition, five of the eight kinked-tailed individuals showed block caudal vertebrae (vertebral fusion, Table S1). Overall, the radiology results demonstrated that the observed kinked-tailed phenotypes in Asian domestic cats is a combined effect of different caudal vertebra deformities including reduction in caudal vertebra number, hemivertebrae, and block vertebrae. The caudal vertebra malformation of kinked-tailed cats in Southeast and East Asia was similar to that of the Japanese Bobtail^20^.

### Linkage analysis of the kinked-tail trait

Genome-wide linkage analysis was performed on the basis of the kinked-tailed cat pedigree. The 13 cats in the pedigree were sequenced to an average depth of 11-fold per individual (Table S2). In total, 29,002,933 SNPs/indels were found, of which 9,413,987 SNPs/indels that covered all of the cats were used for subsequent analysis. We detected 6,656 SNPs/indels that spanned four distinct genome regions on chromosomes B1, B3, C1 and E1 and showed the strongest signal of linkage with short/kinked tails (LOD = 3.01, Fig. 2B). However, the signals detected on cat chromosomes B1 (4 SNPs), B3 (2 SNPs) and C1 (3 SNPs) were the result of three extremely short haplotype blocks (<10 Kb) on each chromosome that were likely to be caused by genome recombination hotspots and thus were discarded as false positives. In contrast, nearly all (>99%) of the significant markers (6,647 SNPs) were located on chromosome E1 and defined a 5.6 Mb haplotype block (ChrE1: 183,971–5,815,703) in complete linkage disequilibrium (LD) with the short-tailed trait in the pedigree, thus representing the true signal of genetic linkage.

### *HES7* is associated with kinked/short tails in Asian cats

**Figure 3:**
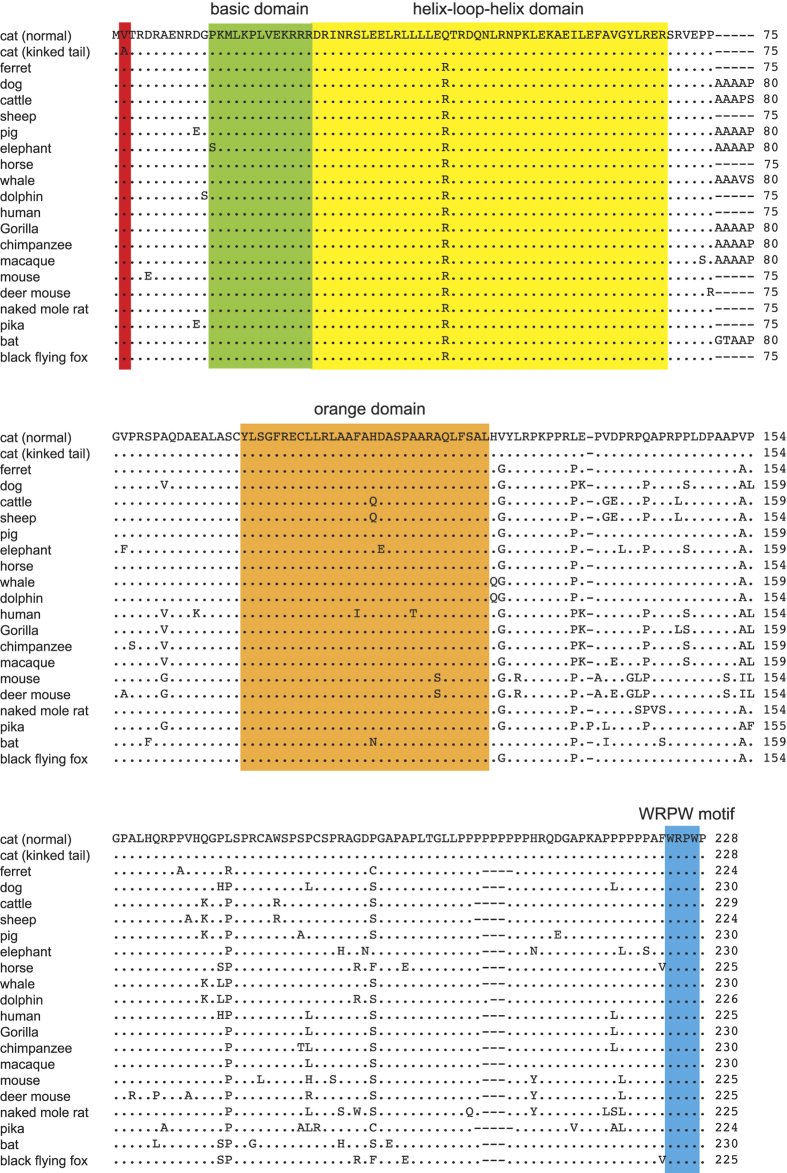
Alignment of amino acid sequences of HES7 among mammals. Dots represent residues identical to the reference sequence of a domestic cat with a wild-type tail phenotype and dashes represent residue gaps in the alignment. The basic domain, the helix-loop-helix domain, the orange domain, and the WRPW motif are marked by green, yellow, orange and blue respectively. The amino acid residue where p.V2A is located is shaded in red.

There were 144 annotated genes in the mapping interval linked with the kinked/short-tailed trait, 11 of which are involved in skeleton development of mice (Table S3). To identify the causal mutation(s), we screened genetic variations, including both SNPs and indels, within the candidate genes on the basis of the whole genome resequencing data of the cat pedigree. While no indel was detected from the coding region of the candidate genes, 2,907 SNPs were identified, of which 68 matched the dominant inheritance model of kinked tails in the pedigree, and were further analyzed. Twelve of the 68 SNPs were non-synonymous substitutions, within which only two (c.2969G > C of *ZBTB4* and c.5T > C of *HES7*) caused amino acid changes at evolutionary conserved residue sites, and were considered as the likely mutations (Table S4). Both candidate variants were tested in 16 kinked-tailed and 16 normal-tailed cats. *ZBTB4* c.2969G > C was not associated with kinked tails (*p* = 0.600) and excluded, whereas *HES7* c.5T > C showed strong signal of association with the kinked-tailed trait (*p* = 3.33E-9), considered as the causal mutation. *HES7* encodes a basic helix-loop-helix oscillatory transcriptional repressor regulating somite segmentation through the Notch pathway^21^,^22^,^23^. Mutations in *HES7* have been shown to be associated with spondylocostal dysostosis (SCD), an axial skeleton development disorder characterized by extensive hemivertebrae and rib anomalies in humans and dogs^24^,^25^,^26^,^27^,^28^, and *HES7*-knockout mice exhibit kinked tail in addition to malformation in the spine and ribs^23^,^28^. The c.5T > C causes a valine-to-alanine missense substitution (p.V2A) at an evolutionarily conserved amino acid residue site in the HES7 protein (Fig. 3). Predictions of the effects of amino acid substitution on protein function by SIFT^29^,^30^ and PolyPhen^31^ suggested that p.V2A may be deleterious to the HES7 protein. Therefore, c.5T > C in *HES7* is probably the causal mutation responsible for the kinked/short tails in Asian domestic cats.

We further validated the *HES7* mutation in an extended sampling of 245 unrelated cats, including 126 feral cats from Asia and 119 breed cats (Table 1). One of the 59 kinked-tailed feral cats from southern China was homozygous for the c.5T > C substitution (C/C), and 38 were heterozygous carrying one copy of the mutant allele (T/C), whereas 20 carried no mutation (T/T). Nevertheless, no mutant was detected among any of the 174 normal-tailed controls, which were all homozygous for the wild-type allele (T/T, Table 1). The results indicate that the *HES7* c.5T > C mutation is strongly associated with short/kinked tails of Asian domestic cats (*p* = 7.821E-30) in accordance with a dominant mode of inheritance, although other mutation(s) might also be involved. We further sequenced the full coding region of *HES7* and *T-box* in the 20 kinked-tailed cats homozygous for the wild-type c.5T allele, but identified no variant associated with their kinked tails, suggesting that there is at least one locus other than *HES7* or *T-box* that leads to the prevalent kinked/short tails in domestic cats from southern China and Southeast Asia. Furthermore, we genotyped the *HES7* mutation in 12 Japanese bobtail cats, all of which were homozygous, thus indicating that the short-tailed trait in the Japanese bobtail is probably fixed with the *HES7* c.5T > C mutation (*p* = 4.014E-19). Notably, individuals homozygous for the *HES7* variant were always of the “extreme kink” category, as observed in both the Asian kinked-tailed cats and the Japanese Bobtail breed, whereas heterozygotes were characterized as either “minor kink” or “medium kink” (Table 1), suggesting a dose effect of *HES7* c.5T > C on tail morphology.

## Discussion

HES7 is a key component of the Notch pathway, which plays an important role in somite formation in vertebrates^21^,^22^,^23^. *HES7* is specifically expressed in presomitic mesoderm (PSM), the unsegmented mesenchyme of the vertebrate embryo, and is directly targeted by the Notch signaling system^21^,^22^,^23^. Repressed by its own encoded protein, *HES7* transcription forms a negative feedback loop, and it oscillates in a 2-hour cycle synchronized with new somite formation in mice^21^,^23^. The transcriptional repression of HES family proteins functions via two mechanisms: 1) HES proteins bind directly to the N-box (CACNAG) through their basic domain and recruit Groucho/TLE family proteins (co-repressor) to the target gene promoter; 2) HES proteins heterodimerize with the E47 protein through their HLH domains, thus preventing E47 from binding to the E-box (CANNTG) and activating transcription^32^.

*HES7* mutations cause spondylocostal dysostosis (SCD), a rare developmental congenital abnormality of the axial skeleton in humans. To date, at least five *HES7* mutations have been identified in human SCD cases, including four missense mutations (p.R25W, p.I58V, p.D142Y, p.D186Y) and one reading frame shift (p.R137QfsX42)^24^,^25^,^26^,^28^. The mutation p.R25W is located within the bHLH domains and impairs the ability of HES7 to bind DNA and form a heterodimer with E47 *in vitro*^25^. Unexpectedly, similar functional defects of HES7 have also been observed in variants caused by missense mutations (p.D142Y and p.D186Y) outside of the bHLH domains *in vitro*, suggesting essential roles of non-bHLH domain residues in maintaining HES7 function^26^,^28^. Although the kinked-tailed variant (p.V2A) found in cats is located upstream of the bHLH domains, it represents an amino acid change at an evolutionarily conserved site and may affect the transcriptional regulation effect of HES7, as observed in the cases of the p.D142Y and p.D186Y mutations in human SCD. Notably, mutations affecting the stability of HES7 also interfere with its normal oscillatory cycle, leading to abnormal somite segmentation in mice^33^. Alternatively, the mutation p.V2A might affect the stability of HES7 by increasing or reducing its half-life, subsequently disrupting the oscillatory cycle of *HES7* transcription. Further functional studies are required to validate the influence of p.V2A on HES7.

In our study, domestic cats with one copy of the *HES7*-p.V2A variant always exhibited kinked tails, although the degree varied, ranging from “minor kink” to “medium kink”, consistently with a dominant mode of inheritance and complete penetrance (Table 1). This result is different from the cases in humans and mice, in which an incomplete penetrance of some *HES7* dominant mutations has been reported^23^,^28^. Additionally, a high rate of infant mortality has been reported for homozygous *HES7* mutants in humans, mice and dogs^23^,^24^,^27^,^28^. Theses mutations either produced reading frame shifts (in humans and dogs) or knockout alleles (in mice) resulting in loss-of-function of HES7^23^,^24^,^27^,^28^. In cats, except for the tail abnormality, feral cats heterozygous or homozygous for p.V2A of *HES7* appear normal and experience no apparent health hazards in urban or rural environments, suggesting that p.V2A may primarily affect the caudal portion of vertebrae only without completely diminishing the function of HES7.

Approximately one-third of the kinked-tailed cats from China examined in this study, all categorized as “minor kink” or “medium kink”, did not carry any of the known genetic variations in *HES7* or *T-box* that are associated with short tails, indicating the existence of additional gene(s) contributing to Asian kinked/short-tailed cats. In our sampling, these kinked-tailed cats with unknown genetic cause were phenotypically similar to the *HES7* p.V2A heterozygotes, including individuals under both the “minor kink” and “medium kink” categories at a similar ratio (p = 0.339, Table 1). Nonetheless, all short-tailed cats categorized as “extreme kink” in our sample set were homozygous for the *HES7* variant. Therefore, there are at least three independent events in the evolutionary history of domestic cats that gave rise to the short- and/or kinked tails. Further studies with extended sampling from Southeast Asia and China will be necessary to elucidate the genetic causes, origins and prevalence of the multiple kinked-tailed mutations in Asian domestic cats.

The Japanese Bobtail is a modern cat breed that was specifically selected for the “bobbed tail” trait, in which the furthest extension of the tailbone from the body should not exceed 10 cm. It was officially registered as a breed in the 1960s in the US, from founders imported from Japan, but before then, naturally occurring short-tailed cats had existed for centuries on the islands of Japan^15^. The domestic cats in Japan were believed to have arrived from China in the early sixth century, probably along with the Buddhist monks who used the cats to keep rats away from their rice paper scrolls^15^. However, whether the “bobbed tail” trait was already present within the originally introduced cats from China or derived from the subsequently thriving cat population in Japan has been unclear. In this study, we demonstrated that the kinked-tailed phenotypes prevalent in southern Chinese street cats and in the Japanese Bobtail are both caused by the *HES7* p.V2A mutation, thus suggesting that the same genetic trait is shared by the two strains.

During the preparation of this manuscript, an independent study has reported that the bobbed tail trait of Japanese Bobtail is associated with the same *HES7* mutation, thus giving further support to the results of this study^34^.

## Materials and Methods

### Pedigree

A cat pedigree segregating for the short/kinked-tailed trait was established by crossing a kinked-tailed female with a normal-tailed male. The female cat was obtained from Guangzhou, Guangdong Province, in southern China and was most probably heterozygous for the short/kinked-tailed trait according to the breeder’s records. The male cat was an American Shorthair purchased from a pet market in Beijing, China. Two litters of 11 kittens were born from the sire and dam (Fig. 2A). When F1 kittens reached the age of three months, tail phenotype examinations were performed for each individual by visual inspection and palpation. Except for the stillborn kitten, all individuals in the pedigree were examined by pelvis and tail radiography at the Veterinary Teaching Hospital, China Agriculture University. The pedigree was maintained at the animal facility of Peking University (PKU) and/or private owners who had adopted cats from the pedigree. All handling of animals and experimental protocols were approved by the PKU Institutional Animal Care and Use Committee (IACUC) and the methods were performed in accordance with the relevant guidelines.

### Sampling and DNA extraction

Whole anticoagulant blood of 12 live cats and a tissue sample from the stillborn kitten were collected from the PKU short-tailed cat pedigree. Buccal samples from unrelated individuals were collected from house or feral domestic cats native in various regions of China, including 59 short-tailed cats from Guangdong and 67 wild-type cats from the provinces of Guangdong (N = 13), Hunan (N = 16), Jiangsu (N = 10), Zhejiang (N = 13), and Jiangxi (N = 5) and the city of Beijing (N = 10). In addition, DNA samples from 119 cats of 21 breeds (Table S5) were provided by the Laboratory of Genomics Diversity, NCI-Frederick, MD, USA, including 12 Japanese Bobtails. Phenotypes of tail length and morphology associated with each sampled cat were visually examined and unambiguously recorded. Blood, tissue and buccal DNA were extracted using a DNeasy Blood and Tissue Kit (QIAGEN) following the manufacturer’s instructions.

### *T-box* gene sequencing

*T-box* mutations have been reported to cause taillessness and/or shorter tail length in the Manx, thus serving as a candidate gene for the short-tailed trait of East and Southeast Asian cats. All of the coding exons of the *T-box* gene were amplified in 20 short-tailed and 20 wild-type cats from southern China. Primers for amplifying segments of *T-box* (Table S6) were designed on the basis of the cat reference genome (*Felis catus* 8.0) using the online software Primer3^35^,^36^. PCR reactions and subsequent sequencing reactions were performed following previously described procedures^37^.

### Whole genome sequencing and SNP calling

Whole genome sequencing was conducted in all 13 individuals from the pedigree at Novogene Corporation, Beijing. For each sample, a sequencing library with average insert size of 250 bp was constructed and loaded into an Illumina Hiseq 2000 sequencer. Approximately 30 Gb of high-quality data in 100 bp pair-end reads was generated, reaching an average of 11-fold genome coverage for each individual. The adaptor sequences at both ends of the reads were trimmed with Cutadapt 1.1^38^. The processed reads were subsequently aligned to the cat reference genome (*Felis catus* 8.0) with the Burrows-Wheeler Aligner 0.6.1 with default parameters^39^. PCR duplications were removed from the alignments. SNP and small indel calling was performed by SAMTOOLS 1.2 according to the standard procedure^40^. SNPs and small indels with low quality (GQ < 20) were filtered.

### Linkage mapping analysis

SNPs and indels covering all 13 cats in the pedigree were selected for genome-wide linkage mapping. Parametric linkage analysis under a dominant model was performed using MERLIN 1.1.2^41^. The nine kinked/short-tailed individuals were set as the affected samples, and the four wild-type individuals were set as the unaffected samples. The disease allele frequency was presumed to be 1% with complete penetrance. A LOD score was calculated for each SNP or indel on the cat’s 18 autosomal chromosomes, and a threshold LOD score was conventionally set to 3 for signals of linkage between an SNP/indel and the kinked/short-tailed trait. Genomic regions with SNPs/indels with LOD scores greater than 3 were selected for further haplotype and linkage analysis of the kinked/short-tailed trait in the cat pedigree.

### Identification of putative causal mutations

Genes located in the linked region of the kinked/short-tailed trait were considered candidate genes. SNPs and indels from the linked region were screened for the putative mutations associated with the short/kinked tail in Asian cats. SNPs/indels from non-coding region were first excluded, and those failing to match the dominant inheritance model in the kinked-tailed cat pedigree were further excluded. SNPs/indels causing amino acid changes from the remaining SNPs/indels within the coding regions were selected and examined for the evolutionary constraints of each affected amino acid residue site. Only the non-synonymous substitutions at evolutionary conserved sites, and indels causing reading frame shifting or affecting conserved amino acid residues were considered to be putative mutations. The possible effects of putative mutations on the corresponding protein were predicted with both SIFT and PolyPhen^29^,^30^,^31^.

### Causal mutation validation

The putative mutations were first tested in 16 kinked- and 16 normal-tailed cats. Mutations showing no signal of association with kinked tails were discarded. The remaining mutation(s) was validated in a large collection of unrelated cats with confirmed tail phenotypes, including 59 kinked- and 67 normal-tailed cats from China and another 119 breed cats (Table S5). Full coding exons of *HES7* and *T-box* were further sequenced in 20 kinked-tailed cats that carried two wild-type alleles at the putative locus. The primer sets to amplify *HES7* exons (Table S6) were designed on the basis of the cat genome assembly (*Felis catus* 8.0). PCR and subsequent sequencing reactions were performed following previously described procedures^37^.

## Additional Information

**Accession codes**: Genome resequencing data have been deposited in the NCBI BioProject database (PRJNA319449, SRP073776), under the accession numbers SRR3436305, SRR3436307, SRR3436357-3436366 and SRR3436368.

**How to cite this article**: Xu, X. *et al*. Whole Genome Sequencing Identifies a Missense Mutation in *HES7* Associated with Short Tails in Asian Domestic Cats. *Sci. Rep.* **6**, 31583; doi: 10.1038/srep31583 (2016).

## Supplementary Material

Supplementary Information

## Figures and Tables

**Figure 1:**
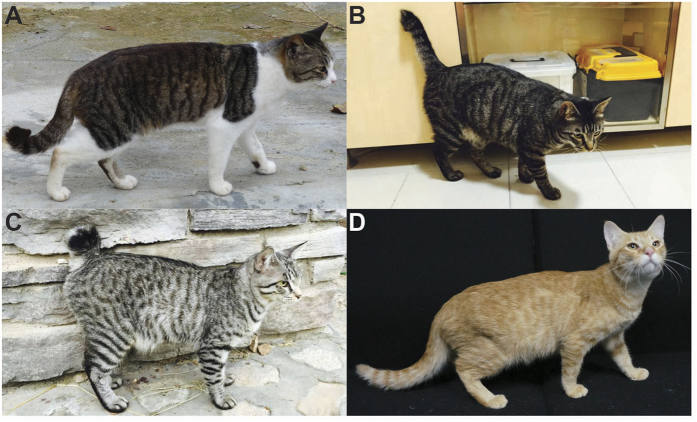
Tail phenotypes of domestic cats from Asia. (**A**) a short/kinked-tailed cat in the “minor kink” category; (**B**) a short/kinked-tailed cat in the “medium kink” category; (**C**) a short/kinked-tailed cat in the “extreme kink” category; (**D**) a cat with a normal wild-type tail.

**Table 1:** Correlation between tail phenotypes and *HES7* genotypes in Asian domestic cats.

Phenotype	N	Genotype (*HES7* c.5T > C)		
		+/+(T/T)	+/mut (T/C)	mut/mut (C/C)
Short/kinked-tailed cats				
minor kink, feral	13	6	7	0
medium kink, feral	45	14	31	0
extreme kink, feral	1	0	0	1
Japanese Bobtail, breed	12	0	0	12
Wild-type cats				
feral	67	67	0	0
breed	107	107	0	0
Total	245	194	38	13
