# Supplementary material for: Whole Genome Sequencing Identifies a Missense Mutation in HES7 Associated with Short Tails in Asian Domestic Cats
Source: Sci Rep. 2016 Aug 25;6:31583. doi: 10.1038/srep31583 (PMC4997960; doi:10.1038/srep31583)
Supplement: Supplementary Information [file srep31583-s1.pdf]

Whole Genome Sequencing Identifies a Missense Mutation in *HES7* Associated with Short Tails in Asian Domestic Cats

Xiao Xu[1], Xin Sun[1], Xue-Song Hu[1], Yan Zhuang[1], Yue-Chen Liu[1], Hao Meng[1], Lin Miao[1], Shu-Jin Luo[1,*]

[1]Peking-Tsinghua Center for Life Sciences, Laboratory of Genomic Diversity and Evolution, School of Life Sciences, Peking University, Beijing 100871, China

* Correspondence should be addressed to S. J. Luo (luo.shujin@pku.edu.cn)

Supplementary Information:

Supplementary Table S1. Radiography results of cats in the pedigree segregating at the kinked/short-tailed trait.

Supplementary Table S2. Statistics of whole genome resequencing data of cats in the pedigree (N=13).

Supplementary Table S3. Genetic variants from the skeleton-related genes within the 5.6 Mb of genomic region linked with kinked/short tails in Asian domestic cats.

Supplementary Table S4. Non-synonymous substitutions linked with kinked/short tails in the cat pedigree.

Supplementary Table S5. Breed cats sample information.

Supplementary Table S6. Primers for *T-box* and *HES7* exons.

Table S1. Radiography results of cats in the pedigree segregating at the kinked/short-tailed trait.

Cat ID	Tail phenotype	No. of caudal vertebrae	No. of caudal hemi-vertebrae	No. of caudal block vertebrae	Relationship
FCAP072	medium kink	14	2	0	dam
FCAP075	normal	22	0	0	sire
FCAP084	medium kink	15	3	1	F1 litter 1
FCAP085	normal	22	0	0	F1 litter 1
FCAP086	normal	22	0	0	F1 litter 1
FCAP087	normal	22	0	0	F1 litter 1
FCAP088	medium kink	15	1	1	F1 litter 1
FCAP089	minor kink	21	2	0	F1 litter 1
FCAP090	medium kink	NA	NA	NA	F1 litter 1
FCAP110	minor kink	21	2	0	F1 litter 2
FCAP111	medium kink	18	1	1	F1 litter 2
FCAP112	medium kink	17	4	2	F1 litter 2
FCAP113	minor kink	19	1	1	F1 litter 2

Table S2. Statistics of whole genome resequencing data of cats in the pedigree (N=13).

Cat ID	Raw bases(Gb)	Unique mapped (Gb)	Unique mapped ratio	WGS Seq depth (unique mapped)
FCAP072	33.402	32.141	96.22%	13.287
FCAP075	29.835	28.426	95.28%	11.751
FCAP084	27.631	26.458	95.75%	10.938
FCAP085	25.948	24.704	95.21%	10.212
FCAP086	31.195	29.808	95.55%	12.322
FCAP087	25.933	24.890	95.98%	10.289
FCAP088	26.483	25.191	95.12%	10.414
FCAP089	25.132	24.068	95.77%	9.950
FCAP090	33.346	32.214	96.61%	13.317
FCAP0110	30.340	28.777	94.85%	11.896
FCAP0111	29.914	28.269	94.50%	11.686
FCAP0112	31.490	29.591	93.97%	12.233
FCAP0113	28.298	26.741	94.50%	11.055
**Average**	29.150	27.791	95.30%	11.488

Table S3. Genetic variants from the skeleton-related genes within the 5.6 Mb of genomic region linked with kinked/short tails in Asian domestic cats.

Candidate Gene	No. SNPs in CDS	No. Synonymous Changes	No. Non-synonymous Changes	No. Linked Non-synonymous Changes	Skeleton phenotype caused by candidate gene mutation(s) in mice
SPNS2	0	0	0	0	increase bone mineral density, increased sacral vertebrae number, decreased lumbar vertebrae number and abnormal rib morphology.
MED31	0	0	0	0	spina bifida, small thoracic cage and small cranium.
DERL2	0	0	0	0	abnormal chondrocyte morphology, abnormal rib morphology, abnormal thoracic cage shape and sternebra fusion.
PFN1	1	1	0	0	abnormal tail morphology, abnormal long bone epiphyseal plate proliferative zone, short femur and short humerus.
TRP53	2	2	0	0	decreased bone mineral density, short humerus and short mandible.
HES7	2	1	1	1	decrease rib number, kinked tail and vertebral fusion.
PER1	4	3	1	0	increased bone mass and increased osteoblast cell number.
TMEM107	0	0	0	0	abnormal basioccipital bone morphology, abnormal exoccipital bone morphology and absent presphenoid bone.
PFAS	24	19	5	0	abnormal cranium morphology and abornal synchondrosis
MYH10	12	12	0	0	abnormal cranium morphology and micrognthia.
MYH1	17	17	0	0	kyphosis.

Table S4. Non-synonymous substitutions linked with kinked/short tails in the cat pedigree.

SNPs position	gene	base change	AA change	evolutionary constraint
ChrE1: 728296	SLC13A5	c.979C>G	p.L327V	unconserved
ChrE1: 781350	KIAA0753	c.1081G>A	p.V361I	unconserved
ChrE1: 892541	FAM64A	c.482G>A	p.R161H	unconserved
ChrE1: 2297535	SPEM1	c.245T>C	p.L82P	unconserved
ChrE1: 2324561	CHRNB1	c.1222C>T	p.P408S	unconserved
ChrE1: 2329714	ZBTB4	c.2969G>C	p.G990A	conserved
ChrE1: 2439792	SOX15	c.463A>G	p.T155A	unconserved
ChrE1: 2863523	HES7	c.5T>C	p.V2A	conserved
ChrE1: 3408840	MFSD6L	c.566G>A	p.R189H	unconserved
ChrE1: 3427381	PIK3R6	c.1819G>A	p.V607I	unconserved
ChrE1: 3436815	PIK3R6	c.882A>C	p.E294D	unconserved
ChrE1: 4116504	USP43	c.2299G>A	p.G767S	unconserved
ChrE1: 4131116	USP43	c.2792G>A	p.R931K	unconserved

Table S5. Breed cats sample information

Breed	Sample No.	Tail phenotype
Japanese bobtail	12	kinked
Abyssinian	4	normal
American Shorthair	4	normal
Bengal	6	normal
Birman	5	normal
Burmese	2	normal
Cornish Rex	2	normal
Devon Rex	12	normal
Havana Brown	8	normal
Egyptian Mau	9	normal
Maine Coon	9	normal
Ocicat	1	normal
Oriental Shorthair	14	normal
Persian	11	normal
Ragdoll	8	normal
Scotish Fold	4	normal
Siamese	3	normal
Somali	1	normal
Sphynx	1	normal
Selkirk Rex	1	normal
Norwegian Forest Cat	2	normal

Table S6. Primers for *T-box* and *HES7* exons.

Amplicon	Size (bp)	Forward primers (5' to 3')	Reverse primer (5' to 3')
T-box-ex1	270	GGGGAAGAGCCTGCAGTA	GGAAGGGAGCAAGAACACC
T-box-ex2	388	CTTCTCTCTGCCCCCACAT	CGGGCTCCATCACACCTAC
T-box-ex3	225	AGGTCTGGTTTCTCTGCTTCC	TCTCTGCATGCTTTGTGAGC
T-box_ex4-5	294	AACCGTGAATTCTTGTTTTTCA	CCGAGACAGAGTGCAACAAA
T-box-ex6	241	AGGCAAATGGCAGTTACTCA	CCGGGTCTACTGCGACTTAG
T-box-ex7	226	TGCCTTTGATGACCAATGAA	GACGCTCTGCATCATGTCC
T-box-ex8	333	CCTGGTTTCTGCCTGACTGT	TTTACGACACAGGCCTCGAT
HES7-ex1	222	CAGGAGCTGCGGGATATAAG	ACACCTCTCCCCTTTCCAAC
HES7-ex2	270	GCCAGCTCTCCCTTTCCT	CTGGGATGGGACTCCAAAG
HES7-ex3	247	CCCACCCCTTTCTGTCTTTG	AGCCTACCGCTCCCCAAG
HES7-ex4	500	GGGCTTCGGTTATCTCTGC	GCCCCGTCTTGTCTGTGC
HES7-ex5	345	GACTGGAGCCGGTAGATCC	GGGCTGGAGTCTCTACCTCA
